# Systematic high-content genome-wide RNAi screens of endothelial cell migration and morphology

**DOI:** 10.1038/sdata.2017.9

**Published:** 2017-03-01

**Authors:** Steven P. Williams, Cathryn M. Gould, Cameron J. Nowell, Tara Karnezis, Marc G. Achen, Kaylene J. Simpson, Steven A. Stacker

**Affiliations:** 1 Tumour Angiogenesis Program, Peter MacCallum Cancer Centre, 305 Grattan St, Melbourne, Victoria 3000, Australia; 2 Ludwig Institute for Cancer Research, Royal Melbourne Hospital, Parkville, Victoria 3050, Australia; 3 Victorian Centre for Functional Genomics, Peter MacCallum Cancer Centre, 305 Grattan St, Melbourne, Victoria 3000, Australia; 4 The Walter and Eliza Hall Institute of Medical Research, Parkville, Victoria 3052, Australia; 5 Sir Peter MacCallum Department of Oncology, University of Melbourne, Parkville, Victoria 3010, Australia; 6 Department of Pathology, University of Melbourne, Parkville, Victoria 3010, Australia

**Keywords:** Collective cell migration, Metastasis, High-throughput screening, Inhibitory RNA techniques

## Abstract

Many cell types undergo migration during embryogenesis and disease. Endothelial cells line blood vessels and lymphatics, which migrate during development as part of angiogenesis, lymphangiogenesis and other types of vessel remodelling. These processes are also important in wound healing, cancer metastasis and cardiovascular conditions. However, the molecular control of endothelial cell migration is poorly understood. Here, we present a dataset containing siRNA screens that identify known and novel components of signalling pathways regulating migration of lymphatic endothelial cells. These components are compared to signalling in blood vascular endothelial cells. Further, using high-content microscopy, we captured a dataset of images of migrating cells following transfection with a genome-wide siRNA library. These datasets are suitable for the identification and analysis of genes involved in endothelial cell migration and morphology, and for computational approaches to identify signalling networks controlling the migratory response and integration of cell morphology, gene function and cell signaling. This may facilitate identification of protein targets for therapeutically modulating angiogenesis and lymphangiogenesis in the context of human disease.

## Background & Summary

The ability of cells to migrate from one location to another within the body is essential for the development of organs and tissues, and for ongoing physiological processes. Cell biologists have studied the migration of cells for decades, and identified a number of the proteins that make up the molecular machinery that drives cells to different locations^1^. Despite this, the contribution and interaction of many gene products to the migratory process, in particular those with cell-type specific roles are not fully defined^2,3^.

Endothelial cells are the specialised cells which line the blood and lymphatic vessels and are required to migrate during embryonic development, and in physiological and pathological circumstances where controlled vessel growth and remodelling is required in the adult (e.g., wound healing and the menstrual cycle)^4–6^. Understanding the molecular signalling that regulates vessel growth and remodelling may enable the development of drugs that modulate these processes, with potential clinical application in human pathologies such as cancer metastasis, lymphoedema and cardiovascular diseases^7–15^.

Recent studies have successfully utilised high-throughput RNAi screening for the identification of genes that are involved in a diverse range of cellular processes such as stem cell differentiation^16^, cancer signalling dependencies^17,18^, ciliogenesis^19^ and sodium channel regulation^20^. Several groups have also used RNAi in screens to specifically identify regulators of cell migration^21–23^. While these screens have provided important insight into the molecular nature of cell migration they have analyzed only a fraction of the genome (6%) in epithelial cells^22^, or <15% in primary blood vascular endothelial cells^23^, or have focused on more generic cell types, e.g., a mouse fibroblast cell line (NIH-3T3)^24^. Importantly, there have been no such RNAi-based functional studies on the migration of lymphatic endothelial cells.

**Figure 1:**
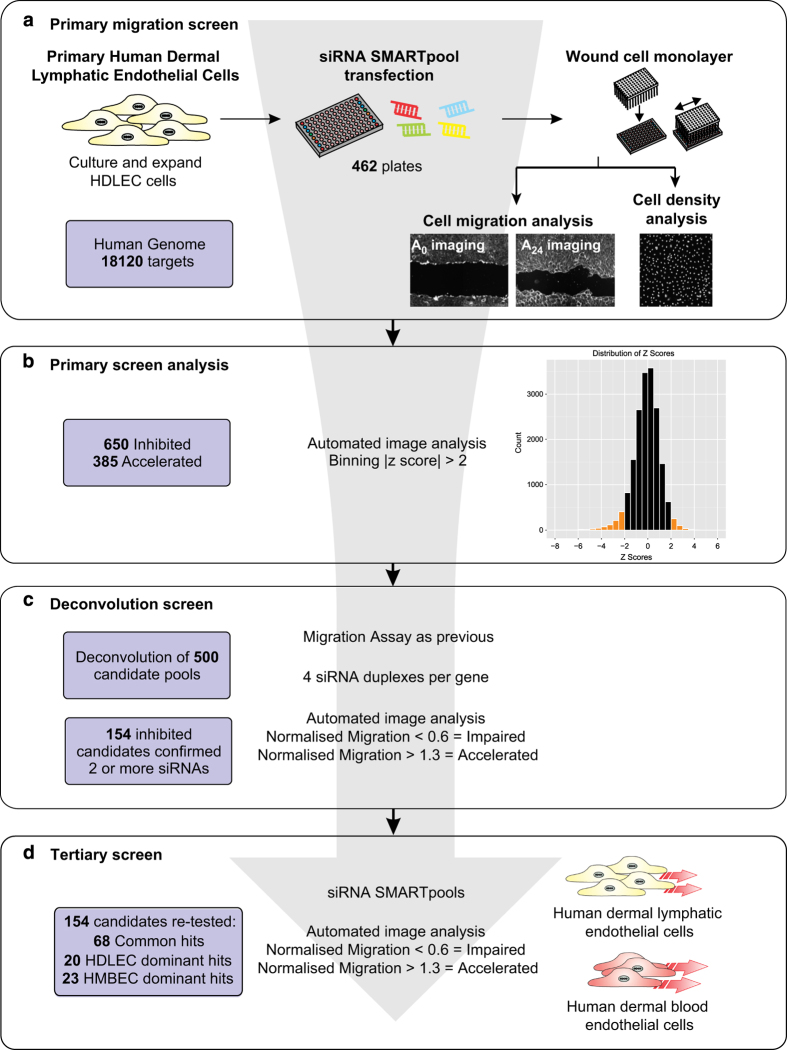
Overview of the successive siRNA migration screens. (**a**) Experimental scheme for the genome-wide siRNA screen to identify genes required for cell migration. HDLEC cells were reverse transfected with siRNA SMARTpools 48 h prior to assessment for migratory capacity in the monolayer scratch assay. Image based analysis was used to calculate a migration score and a cell density score. The primary screen assessed siRNA SMARTpools targeting 18,120 protein-coding genes (see Data Record 1). (**b**) Automated image analysis was used firstly for cell nuclei count; samples that were identified as ‘Low Cell Density’ were excluded from migration analysis. Image analysis was then used to measure cell migration (Data Record 4). Results with migration |robust z score|>2 were considered as candidate hits. (**c**) The deconvolution screen confirmed the phenotypes of 154 genes as regulators of LEC migration (see Data Record 2). (**d**) A tertiary screen was performed to compare and contrast the role of confirmed migration regulators in two dermal endothelial cell types: HDLECs and HMBECs (Data Record 3). Morphological analysis of gene knockdown phenotypes was also performed in order to identify genes with similar functional roles (Data Records 5 and 6).

Here we describe a dataset of successive high-throughput siRNA screens which were used to identify regulators of lymphatic endothelial cell migration (the overall experimental design is shown in Fig. 1). The primary and secondary screens were performed using primary human dermal lymphatic endothelial cells (HDLECs). The tertiary screen aimed to compare and contrast migration in lymphatic endothelial cells with human microvascular blood endothelial cells (HMBECs), to better understand the common and unique signalling molecules utilised by each cell type.

Following incubation with a genome-wide siRNA library, cells were assessed for the ability to migrate and close a scratch wound in the endothelial cell monolayer. In total, 650 siRNAs were binned as 'Inhibited' migration (decreased migration ability), and 385 siRNAs caused 'Accelerated' (enhanced) migration (Data Record 1). A further 438 siRNAs were scored as 'Low Cell Count' (decreased viability).

We selected 500 candidate migration hits (401 ‘Inhibited’ and 99 ‘Accelerated’) for confirmation in a secondary deconvolution screen. One hundred and thirteen medium confidence and 41 high confidence genes were identified as causing 'Inhibited' migration following siRNA knockdown (Table 1 and Data Record 2). Further, 22 genes were classed as causing non-specific toxicity, as 1/4 siRNA caused a ‘Low Cell Count’ (no phenotype seen with the other three siRNAs). Of the ‘Accelerated’ candidates, only 5 of the 99 scored 1/4 siRNA as ‘Accelerated’, while the remainder did not reproduce the original phenotype; ‘Accelerated’ candidates were therefore not investigated further.

A comparative tertiary siRNA screen was used to assess the importance of candidate hits in HMBECs. This enabled 68 genes to be assigned to ‘core endothelial migration genes’, 23 genes as ‘BEC dominant migration genes’ and 20 as ‘LEC dominant migration genes’ (Data Record 3).

The process of cell migration is closely linked to changes in cell morphology, as the actin cytoskeleton is remodelled to propel the cell forward. We employed high-content image analysis to explore the morphological changes induced by knockdown of candidate genes in the context of migrating cells. This analysis enabled a morphological signature to be assigned to each siRNA gene knockdown (Data Record 5). For a novel candidate gene that is relatively unstudied, the morphology signature may give clues as to the candidate’s functional role in migration and cytoskeletal signalling.

The datasets presented here will be of benefit to the fields of cell migration, endothelial biology, molecular signalling and broadly provides the high throughput screening community with a robust method and screening dataset. Furthermore, the datasets will facilitate identification of molecular targets for novel therapeutics designed to modulate the remodelling of lymphatics and blood vessels in cancer, cardiovascular diseases and inflammatory conditions.

## Methods

### Cell lines

Human neonatal dermal lymphatic microvascular endothelial cells (HDLEC) (single donor, #CC2812) and human neonatal dermal blood microvascular endothelial cells (HMBEC) (single donor, #CC2813) (Lonza, Basel, Switzerland) were grown using EBM2 media with EGM-2-MV Singlequots supplements (Lonza) and 10 mM HEPES (Gibco, Life Technologies, Grand Island, NY). A single batch of cells was used throughout the entire screen. Cells were cultured on tissue culture plates coated with 5 μg ml^−1^ human fibronectin solution (BD Bioscience, San Jose, CA).

### High throughput RNA interference screening

The protocol developed for this screen was modified from previously published methods^22,25^, and is described below in detail. The primary siRNA screen was performed in technical duplicate, in 96-well plates. Positive and negatives controls were located in columns 1 and 12, as depicted in Supplementary Fig. 1. The positive controls were siRNA SMARTpools targeting CDC42 (GE Dharmacon, #M-005057-01-0020); known to be important for cell migration, and targeting CDH5 (Vascular Endothelial cadherin) (GE Dharmacon, #M-003641-01-0020); a key component of endothelial cell-cell junctions. Mock transfected (lipid only) wells were used as the negative control. At the time of screening, all non-targeting control siRNAs tested were found to be sub-optimal as they affected the cellular phenotype (Non-targeting siRNA #2 (GE Dharmacon, #D-001210-02-20) was included as a reference).

The siRNA transfection step was performed using a SciClone ALH3000 Lab Automation Liquid Handler (Caliper Lifesciences). An EL406 Microplate Washer Dispenser (BioTek) was used for all other liquid aspirating and dispensing steps. High content imaging of the scratch assay was carried out on a Pathway 435 imaging platform (BD), while imaging of the cell nuclei count and morphology assays was performed on a Cellomics VTI Arrayscan HCS System (Thermo Fisher Scientific), with attached plate handler (Twister II, Caliper Life Sciences).

Specialised consumable reagents that were required to perform the siRNA screens:

- Dharmacon siGENOME siRNA SMARTpool library RefSeq27. This corresponds to 231×96-well plates.
- DharmaFECT 1 Transfection Reagent (GE Dharmacon, #T-2001-04).
- Opti-MEM (Gibco, #31985062).
- 96-well plate, polystyrene, tissue-culture treated, clear flat bottom wells, sterile, with lid, black (Corning, #CLS3603).
- Celltracker Green CMFDA (Life Technologies, #C2925); final concentration 5 μM (1:2000).
- Hoechst 33342 (Life Technologies, #H3570); 20 μg ml^−1^ (1:500).
- Phalloidin CF488 (Biotium, #00042); 20 nM (1:300).

Reagents required for morphology analysis:

- Hoechst 33342 (Life Technologies, #H3570); 20 μg ml^−1^ (1:500).
- Phalloidin CF555 (Biotium, #00040); 20 nM (1:300).
- Celltracker Green CMFDA (Life Technologies, #C2925); 5 μM (1:2000).

### Day 1: Reverse transfection

siRNA library and control plates were thawed at room temperature 1 h prior to use. Cells were incubated in 0.25% Trypsin-EDTA (Gibco, #25200-072) for 5 min until detached, washed in growth media, counted and diluted to 187,500 cells per ml.

For the primary screen the siRNA library was screened once, using technical replicates of each plate (‘A’ and ‘B’). For each siRNA library plate, DharmaFECT 1 transfection reagent was diluted in Opti-MEM at a ratio of 1 μl:79 μl and incubated for 5 min, before 32 μl were dispensed into the ‘A’ replicate assay plate (Biotek 406 multiwell dispenser).

Next, 8 μl of the siRNA library (1 μM stock) were dispensed into lipid/Opti-MEM in plate ‘A’ (using a SciClone liquid handling robot), and mixed well. From this mixture, 20 μl was transferred to assay replicate plate 'B’, thus ensuring both replicate assay plates were derived from the same transfection mix. Assay plates were incubated for 20 min at room temperature, before dispensing 80 μl cells (15,000 cells) to each well (BioTek 406 multiwell dispenser) (total well volume 100 μl. Final siRNA concentration 40 nM). The plate layout was designed so that control wells (positive and negative) were distributed in columns 1 and 12 (Supplementary Fig. 1).

The secondary deconvolution validation screen was performed once, using technical replicates of each plate (‘A’ and ‘B’). Final siRNA concentration was 25 nM. Briefly, 14 μl of individual siRNA duplexes (4 per SMARTpool; 0.45 μM) were picked and arrayed into individual wells of 96-well source plates (Supplementary Fig. 1). DharmaFECT 1 reagent was diluted in Opti-MEM media at 1 μl:79 μl, mixed well and then incubated at room temperature for 5 min, before 36 μl were dispensed into each well of every source plate containing siRNAs. The DharmaFECT/siRNA mixture was mixed well, and 20 μl transferred to each of assay replicate plates 'A' and 'B'. Plates were then incubated for 20 min at room temperature to allow formation of the DharmaFECT/siRNA complex. Cells were then dispensed into assay plates as described above.

The tertiary re-screen was performed in biological duplicate, each with technical replicates of each plate (‘A’ and ‘B’). The tertiary screen utilised siRNA SMARTpools (final concentration 40 nM). The transfection protocol was identical to the primary screen. The assay plate layout is shown in Supplementary Fig. 1.

### Day 2: Media change

Media was changed on all assay plates 24 h after transfection using a BioTek 406 liquid handling robot. Aspiration steps were performed using the following parameters: z=36 (4.57 mm above carrier), x=0, y=26 (1.92 mm front of centre), to ensure that the cell monolayer was not disturbed. In order to overcome any toxicity that may arise from residual transfection lipid remaining in the wells, a large volume of prewarmed fresh growth medium (100 μl) was added to each well. Plates were incubated for a further 24 h.

### Day 3: Scratch wound migration assay

At 47 h post-transfection, media was removed and replaced with growth media containing Celltracker Green CMFDA live whole-cell dye (5 μM; Invitrogen). Plates were then incubated at 37 °C for 45 min to allow dye incorporation. At 48 h post-transfection, the confluent cell monolayer was wounded using a V&P Scientific wounding replicator using FP pins that delivered a precise scratch of 0.38×3.8 mm (www.vp-scientific.com/wounding_tissue_culture_experiments.php) driven using a Sciclone ALH 3000 workstation robot (Caliper Life Sciences, Hopkinton, MA). Wells were washed once with PBS, and refilled with 100 μl normal growth media, and an image of the initial scratch area (A_0_) was then obtained (Data Record 4) as described in High Content Imaging, before incubating at 37 °C for a further 24 h. Note that for the ‘Remaining Genome’ subset of the siRNA library, an image of the initial scratch area (A_0_) was only obtained for one plate per batch (an extra plate, named the ‘Sentinel plate’, was specially seeded for this purpose). This was possible as the scratch dimensions produced by each scratch pin were consistent (Coefficient of variation (CV) of scratch area=11.6%; CV of scratch width=13.3%). Using a ‘Sentinel plate’ reduced processing time and reagent volume used.

### Day 4: Endpoint

At 72 h post-transfection, cells were fixed using 4% PFA (20 mins at room temperature) and stained with phalloidin CF488 (or phalloidin CF555 for Tertiary screen plates) (20 nM, Biotium, Hayward, CA) and Hoechst 33342 (2 μg ml^−1^, 40 mins at room temperature). Cells were then washed once with PBS, and refilled with 100 μl PBS, so that excess dye was removed from the well. An image of the remaining scratch area (A_24_) was obtained as described in ‘High content imaging of cell migration’ (Data Record 4).

### High content imaging of cell migration

A high-throughput imaging system (Pathway 435, BD Bioscience) was used to capture fluorescent images of cells. A 4× UPlanSApo NA0.16, or 20× UApo NA0.75 objective (Olympus, Tokyo, Japan) was used with 2×2 pixel binning (4× objective: 2.97 μm per pixel; 20× objective: 0.59 μm per pixel). High-content acquisition software (Attovision v1.6.1, Becton Dickinson) enabled capture of two (or more) adjacent fields of view, which were then stitched together as a montage image. Images were flat-field corrected on capture.

Image files were subjected to an analysis protocol established in image analysis software (MetaMorph v7.7.5.0 (64-bit), Molecular Devices, Sunnyvale, CA) using several image processing steps: a center-filter step was performed to smooth the image, and then the background was flattened. The resulting image was auto-thresholded for light objects; the threshold was used to create a black and white binary image of the wound area. The binary wound area was dilated by 4 pixels to ensure that the measured area did not include cells, and then inverted. This allowed the wound area not covered by cells to be accurately measured, and ensured that debris and small tears were not measured, regions with <10,000 pixels were excluded from A_0_ images, and regions <100 pixels were excluded from A_24_ images.

Images of all scratch wounds were examined by eye using the low resolution ‘plate view’ of the Attovision acquisition software, and excluded for reasons such as failure to scratch the monolayer, excess debris, contamination or poor cell seeding. Images of the scratch (A_0_ and A_24_) were analysed, and the resulting data used to calculate the area migrated over (Am) by cells during the assay period. The migration of the cells was described by:

$$\mathrm{Am}=\mathrm{Initial}\;\mathrm{area}\;\mathrm{of}\;\mathrm{wound}(A_{0})-\mathrm{Wound}\;\mathrm{area}\;\mathrm{remaining}(A_{24}).$$

Each well was normalized to the median of Mock-transfected negative control wells to obtain a normalized migration score. The average of technical replicate plates was taken and robust z-scores (utilizing the median and median absolute deviation (MAD)) were calculated. Active siRNAs were identified using |robust z score|>2, and binned as ‘Inhibited’ (robust z score<−2) or ‘Accelerated’ (robust z score >2) (Data Record 1).

In the Secondary deconvolution screen, active siRNAs were identified by the normalized migration score. For each plate, Am values were normalized to the median Mock-transfected control Am value. The average of technical replicate plates was taken, and active siRNAs were binned as ‘Accelerated’ when the normalized migration score >1.3, and binned as ‘Impaired’ when normalized migration<0.6. These binning thresholds corresponded closely to the mean of mock-transfected control wells±3 s.d.'s (Data Record 2). Medium confidence was assigned to genes where 2/4 siRNAs reproduced the original phenotype, and high confidence where 3/4 or 4/4 siRNAs reproduced the original phenotype.

### Cell nucleus count viability assay

Effects on cell viability were detected by imaging and counting of cell nuclei using a high-content imaging system (Cellomics VTI Arrayscan, Thermo Fisher Scientific, Pittsburgh, PA) with an attached plate handler (Twister II, Caliper Life Sciences). Acquisition software (Cellomics Scan software v7.6.2.1, Thermo Fisher Scientific) was used to capture images using a 10× Plan-NeoFluar NA0.3 objective (Carl Zeiss, Oberkochen, Germany), with 30 adjacent fields imaged. A ‘TargetActivation v4’ bioapplication was used with thresholding to identify valid nuclei, which included all objects with total area between 60 and 1,400 pixels, and with average intensity less than 4,095. The median cell density at the migration assay endpoint was 187.5 cells/field. An arbitrary cut-off of 110 cells/field (<60% of median) was established for defining wells as having a low cell density; these wells were visually distinct from mock-transfected wells. Cell nuclei count binning was given priority over cell migration binning, and consequently all siRNAs binned as ‘Low Cell Count’ were excluded from further migration analysis.

### Morphology image analysis

Fluorescent images were captured by an Cellomics VTI Arrayscan imaging system as described above, using a 20× UApo NA0.75 objective (Olympus) with 2×2 pixel binning (20× objective: 0.59 μm per pixel) (Data Record 6). A montage of 2×3 adjacent fields was automatically captured. Cell morphology was then assessed by segmenting images using the multi-wavelength cell scoring application module in MetaMorph v7.7.10 (Molecular Devices). Hoechst and Celltracker Green CMFDA images were used for nuclear and whole cell segmentation respectively. The resulting whole cell masks were used to generate binary masks. These masks were then used in conjunction with the Integrated Morphometric Analysis module in MetaMorph to measure the following parameters from the phalloidin stained image: area, perimeter, breadth, length, shape factor, elliptical form factor, texture difference moment, inverse texture different moment, average intensity, total intensity and intensity s.d. The average of replicate plates was taken for each parameter and then z-score normalized (Data Record 5).

The dataset was clustered using CIMminer (http://discover.nci.nih.gov/cimminer/), using the following settings: Correlation distance algorithm, Complete linkage cluster method, and Quantile binning. A dendrogram height of 1-Pearson correlation=1.5 used as the cut point for the dendrogram tree. Each cluster was assigned a number, which is reported for each gene in the cluster (Data Record 5).

## Data Records

### Data record 1

Primary siRNA screen data are available at PubChem under the accession number AID 1159578 (Data Citation 1). We provide screen-wide normalised data, robust z-scores, and the results of binning strategies. The PubChem activity score indicates whether a siRNA SMARTpool was ‘active’ (designated 1, i.e., a screen hit) or ‘inactive’ (designated 0, i.e., not a screen hit). Samples are defined by siRNA catalogue number (Thermo Fisher) and Entrez Gene ID.

### Data record 2

Secondary deconvolution siRNA screen data are available at PubChem under the accession number AID 1159579 (Data Citation 2). We provide screen-wide normalised data, and the results of binning strategies. PubChem activity score and sample definition are as Data record 1 above.

### Data record 3

Tertiary siRNA screen data are available at PubChem under the accession number AID 1159618 (Data Citation 3). We provide screen-wide normalised data for both cell types, and the results of binning strategies. Sample definitions are as Data record 1 above.

### Data record 4

Images of HDLEC primary screen scratch assays are available at Figshare with the doi:10.6084/m9.figshare.3980184 (Data Citation 4).

### Data record 5

Tertiary siRNA screen morphology data for both cell types are available at PubChem under the accession number AID 1159617 (Data Citation 5). Screen-wide robust z-scored data is provided, as well as the results of unsupervised hierarchical clustering. PubChem activity score and sample definition are as Data record 1 above.

### Data record 6

Images of both migrating HDLEC and HMBEC that were used for morphology analysis are available at Figshare with the doi:10.6084/m9.figshare.c.3150637 (Data Citation 6).

## Technical Validation

### Reproducibility across screening experiments

**Figure 2:**
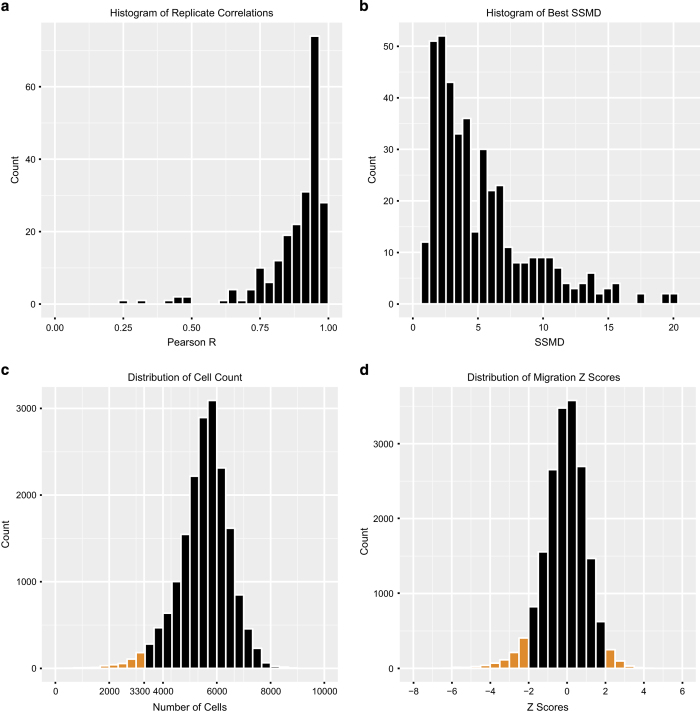
Distribution of primary screen metrics. (**a**) Distribution of Pearson R values for correlation between technical replicate plates. (**b**) Distribution of Strictly Standardised Mean Difference (SSMD) scores, a measure of control siRNA performance and plate QC. (**c**) Distribution of cell nuclei count for siRNA treated cells. A threshold of 3300 nuclei was used for binning as ‘Low Cell Count’ (coloured in orange). (**d**) Distribution of siRNA migration z-scores, with hits |robust z score |>2 coloured in orange.

The genome-wide siRNA library was screened once, with technical replicates of each plate (‘A’ and ‘B’). Technical replicate plate reproducibility was calculated for each set of duplicate plates using the Pearson correlation coefficient. Replicate ‘A’ and ‘B’ plates that passed quality control (QC) (described below) were highly correlated with each other (median=0.92). The distribution of Pearson correlation coefficients between replicate plates is presented in Fig. 2a.

### Control performance and plate QC

Positive and negative control performance in the primary screen was assessed by the Strictly Standardised Mean Difference (SSMD)^26–29^, a statistical measure of the difference between positive and negative controls, taking into account the magnitude of the difference, as well as the variation in the control populations. Generally, an acceptable SSMD for an RNAi screen is≥1 in the context of a moderate positive control^26,28,29^.

As discussed above, Mock-transfected wells were used as negative controls. A Non-targeting siRNA #2 was included as a reference in the screen, but the migration in these wells was accelerated (compared to both Mock-transfected wells, and to the median of siRNA library wells; see Supplementary Fig. 2) and therefore was not utilised in analysis of the screen.

SSMD was calculated for each plate using both positive controls (siCDC42 and siCDH5). For each plate the highest SSMD value was used to pass or fail a plate. Plates with SSMD≥1 were passed (Fig. 2b). Plates that passed this QC criteria had an average MOCK/siCDC42 SSMD of 4.12 and median of 3.12, and an average MOCK/siCDH5 SSMD of 3.60 and median of 2.49. This indicates the quality of the screen data is ‘excellent’ in the context of a moderate positive control^26,29^. Eight assay plates were failed, so that only a single technical replicate was available for these library plates. For 19 plates it was not possible to calculate SSMD scores, as only a single well of each positive control was usable (due to logistical issues e.g., well contamination) (Plates 11184–11192, replicates ‘A’ and ‘B’, and plate 11087 ’B’).

### Primary screen identified known migration candidates

The primary screen cell count assay identified many genes known to be important for cell viability and proliferation (Data Record 1). The cell nuclei count was used to identify 438 siRNAs that reduced the cell viability (2.4% of library). The distribution of cell count values is shown in Fig. 2c. A threshold of 3300 (<60% of median) was set for binning. These genes were binned as ‘Low Cell Count’ and excluded from migration analysis. Examples include VEGFR3 (a critical receptor for lymphangiogenic growth factors), cell cycle molecules such as PLK1, WEE1, CDK1, CDK4, Cyclin A2, Cyclin B1, Cyclin D1 and the pro-survival protein MCL1.

**Figure 3:**
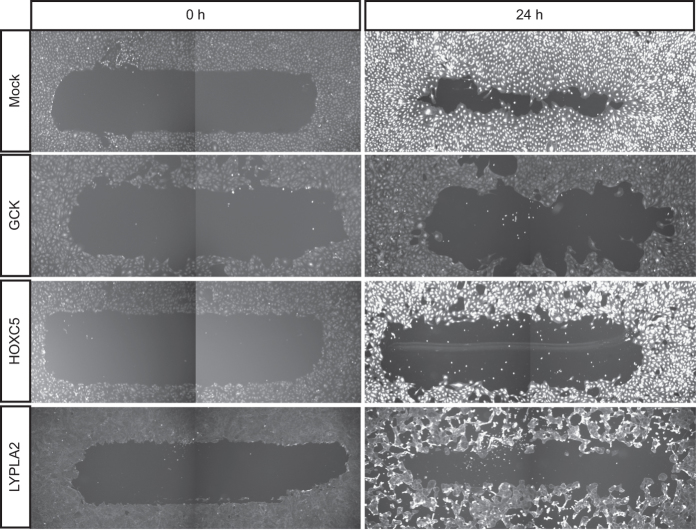
Examples of Mock-transfected migration, and siRNAs that were scored as impaired HDLEC migration from the primary siRNA screen. The cells were stained with Celltracker Green CMFDA for the image captured at 0 h, and then fixed and stained with Phalloidin CF488 for the image captured at 24 h.

The distribution of migration z scores for the primary screen is shown in Fig. 2d. A cut off of |robust z score|>2 was used to identify siRNAs that significantly affected HDLEC migration. Examples of ‘Impaired’ migration are shown in Fig. 3. The primary screen successfully identified genes that have been previously shown to regulate cell migration (Data Record 1). Among the top hits were CDC42 and the downstream effector kinase LIMK1, and the Rho effector Rhotekin (robust z scores=−2.46, −2.39 and −2.34 respectively), all previously identified as regulators of cell migration^6,30,31^. In addition, the screen identified important endothelial receptors such as PDGFRB (robust z score=−2.44), and the ligand ANGPT2 (robust z score=−2.93)^32,33^. These results confirmed the ability of the screening methodology to identify genes involved in cell migration and endothelial biology. Characterisation of novel hits will be the subject of further publications.

### Numbers of genes validating in the deconvolution screen

Table 1 summarises the frequency of genes validating with different numbers of siRNA duplexes for each assay in the deconvolution screen. Analysis of the deconvolution screen data (Data Record 2) resulted in the validation of 30.8% of candidate genes with medium or high confidence (0.6% with 4/4 siRNAs, 7.6% with 3/4, and 22.6% with 2/4). Of these, 114 ‘Impaired’ genes were validated with medium confidence (2/4 duplexes), 38 genes with high confidence (3/4) and three genes with very high confidence (4/4). ‘Accelerated’ phenotypes were not validated in this assay. This rate of validation is similar to that seen in other genome-wide siRNA screens^19,20,34^.

### Numbers of genes validating in the tertiary screen

The tertiary screen aimed to compare the blood and lymphatic endothelial cell types. siRNA SMARTpools targeting the 154 genes identified as medium-high confidence hits in the secondary screen were rescreened in both HDLECs and HMBECs. Analysis showed 111 genes scored impaired migration in at least one of the two cell types (Data Record 3). Of these, 68 (61.3%) impaired the migration of both cell types. Candidate genes were grouped into 68 ‘Common migration genes’ that were important for the migration of both cell types, 23 ‘BEC dominant genes’ that caused a greater effect on HMBEC migration than on HDLECs, and 20 ‘LEC dominant genes’ that caused a more pronounced effect on HDLEC migration than on HMBECs (Data Record 3). These hits represent highly validated migration genes and warrant further investigation, particularly in order to better understand the cell type specific signalling requirements for the ‘BEC’ or ‘LEC dominant genes’.

### Morphology analysis

In order to investigate morphological changes that occurred following siRNA-mediated gene knockdown, we utilised high content imaging to capture and analyse fluorescent microscopy images of migrating cells (Data Record 6). Both positive control siRNAs (siCDC42 and siCDH5) that were included in the migration screens also induced visible changes in HDLEC morphology. The image-based morphology analysis was able to detect this and scored siCDC42 with z-score >2 for Elliptical form factor and z-score<−2 for Shape factor, corresponding to an elongated phenotype (Data Records 5 and 6). siCDH5 scored >2 for Total Actin Intensity, corresponding with the bright accumulation of actin observed as these cells detached from one another. In contrast, Mock-transfected cells did not score highly in any of the parameters measured.

**Figure 4:**
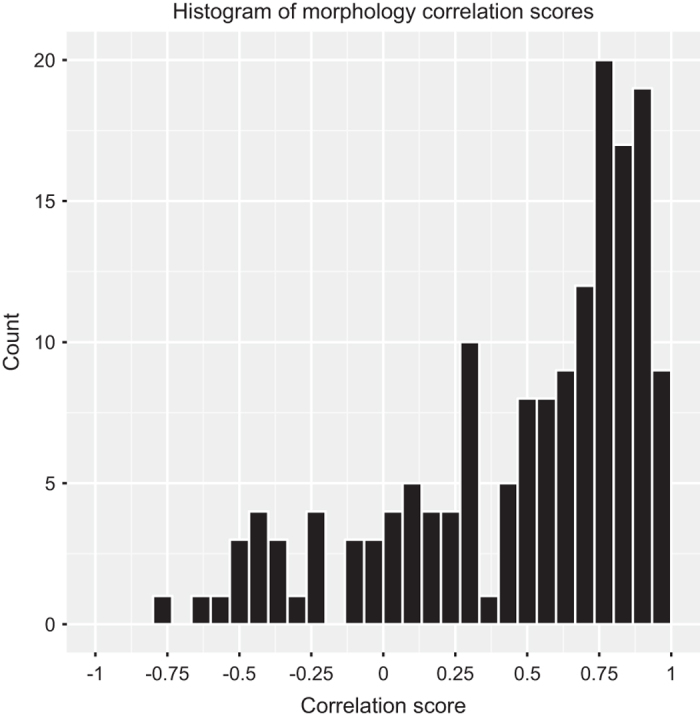
Morphology phenotype correlations between HDLEC and HMBEC. The distribution of Pearson correlation values for the morphology signatures of HDLEC and HMBEC treated with siRNAs in the Tertiary screen is shown.

The HMBEC morphology signatures following gene knockdown largely resembled that seen in HDLECs (Data Record 5), with some intriguing differences that may represent differences in the molecules that regulate actin remodelling and cellular shape in these specific cell types. However, overall the morphology signatures induced by siRNA knockdown correlated strongly between the two cell types (Pearson R median=0.65) (Fig. 4), further supporting the finding that many of the validated hits from the Tertiary screen are ‘Common endothelial migration’ genes (Data Record 3).

To investigate whether cell migration and morphological features were correlated, we plotted normalised cell migration scores against the z-scores for each feature (Supplementary Figs 3 and 4). Interestingly, while there are no strong correlations in this dataset, F-actin Intensity Std. Dev. appears to be linked to the migration of HDLEC (Supplementary Fig. 3). HDLEC treated with siRNAs that strongly impaired migration show higher F-actin Intensity Std. Dev. scores (e.g., the transcription factors *HOXC5* and *ZNF76*), suggesting that these targeted genes may play a role in coordinating the formation or remodelling of actin filaments for migration. HMBEC migration appears to also be correlated with F-actin Intensity Std. Dev. to a small extent, but also to cell size (Supplementary Fig. 4). siRNAs that strongly impaired migration in HMBEC (e.g., *PMVK* and *S1PR*) have larger area, length and breadth, suggesting that targeting these genes may cause cellular remodelling to be disconnected from cell migration. However, this phenotype is not so apparent in HDLEC, pointing to possible differences in migration signalling pathways.

## Usage Notes

The siRNA screening data (Data records 1–3) are provided for users to be able to apply their own normalisation strategies and thresholds for identification of migration screen candidate hits, as well as analysis of genes that are essential for endothelial cell viability and proliferation. This study focused on hits that impaired cell migration as a way to understand the unwanted lymphangiogenesis and lymphatic vessel remodelling seen in human pathologies such cancer. The proteins encoded by the identified genes could represent potential anti-lymphangiogenic therapy targets. However, 385 siRNAs produced an Accelerated phenotype in the primary screen, and may therefore also be important positive regulators of cell migration. These could be important when considering molecular mechanisms to promote lymphangiogenesis or remodelling in diseases such as lymphedema where lymphatic vessel formation is deficient.

Migration images (Data record 4) are provided for users to be able to apply their own image analysis protocols to assess cell migration phenotypes and identify further migration screen candidate hits.

Morphometric data is provided (Data records 5 and 6) for users to investigate morphologies induced by siRNA gene knockdown and their potential correlation with other signalling pathway perturbations and the actions of existing drugs or targeted therapies.

## Additional Information

**How to cite this article:** Williams, S. P. *et al.* Systematic high-content genome-wide RNAi screens of endothelial cell migration and morphology. *Sci. Data* 4:170009 doi: 10.1038/sdata.2017.9 (2017).

**Publisher’s note:** Springer Nature remains neutral with regard to jurisdictional claims in published maps and institutional affiliations.

## Supplementary Material



Supplementary Information

## Figures and Tables

**Table 1:** Binning results of secondary siRNA screen.

	**Binning**						
	**Toxic**	**0/4**	**1/4**	**2/4**	**3/4**	**4/4**	**Total**
**Impaired**	21	79	147	113	38	3	401
**Accelerated**	1	92	6	0	0	0	99
**Percentage**	4.4%	34.2%	30.6%	22.6%	7.6%	0.6%	100.0%
