# Supplementary material for: Systematic high-content genome-wide RNAi screens of endothelial cell migration and morphology
Source: Sci Data. 2017 Mar 1;4:170009. doi: 10.1038/sdata.2017.9 (PMC5332011; doi:10.1038/sdata.2017.9)
Supplement: Supplementary Information [file sdata20179-s2.pdf]

Primary screen plate layout

	1	2	3	4	5	6	7	8	9	10	11	12
A	Mock	siRNA SMARTpools	siRNA SMARTpools	siRNA SMARTpools	siRNA SMARTpools	siRNA SMARTpools	siRNA SMARTpools	siRNA SMARTpools	siRNA SMARTpools	siRNA SMARTpools	siRNA SMARTpools	Mock
B	Non-targeting #2	siRNA SMARTpools	siRNA SMARTpools	siRNA SMARTpools	siRNA SMARTpools	siRNA SMARTpools	siRNA SMARTpools	siRNA SMARTpools	siRNA SMARTpools	siRNA SMARTpools	siRNA SMARTpools	Non-targeting #2
C	Non-targeting #2	siRNA SMARTpools	siRNA SMARTpools	siRNA SMARTpools	siRNA SMARTpools	siRNA SMARTpools	siRNA SMARTpools	siRNA SMARTpools	siRNA SMARTpools	siRNA SMARTpools	siRNA SMARTpools	Non-targeting #2
D	si_CDC42	siRNA SMARTpools	siRNA SMARTpools	siRNA SMARTpools	siRNA SMARTpools	siRNA SMARTpools	siRNA SMARTpools	siRNA SMARTpools	siRNA SMARTpools	siRNA SMARTpools	siRNA SMARTpools	si_CDC42
E	si_CDH5	siRNA SMARTpools	siRNA SMARTpools	siRNA SMARTpools	siRNA SMARTpools	siRNA SMARTpools	siRNA SMARTpools	siRNA SMARTpools	siRNA SMARTpools	siRNA SMARTpools	siRNA SMARTpools	si_CDH5
F	Mock	siRNA SMARTpools	siRNA SMARTpools	siRNA SMARTpools	siRNA SMARTpools	siRNA SMARTpools	siRNA SMARTpools	siRNA SMARTpools	siRNA SMARTpools	siRNA SMARTpools	siRNA SMARTpools	Mock
G	Non-targeting #2	siRNA SMARTpools	siRNA SMARTpools	siRNA SMARTpools	siRNA SMARTpools	siRNA SMARTpools	siRNA SMARTpools	siRNA SMARTpools	siRNA SMARTpools	siRNA SMARTpools	siRNA SMARTpools	Non-targeting #2
H	Mock	siRNA SMARTpools	siRNA SMARTpools	siRNA SMARTpools	siRNA SMARTpools	siRNA SMARTpools	siRNA SMARTpools	siRNA SMARTpools	siRNA SMARTpools	siRNA SMARTpools	siRNA SMARTpools	Mock

Secondary deconvolution screen plate layout

	1	2	3	4	5	6	7	8	9	10	11	12
A	Mock	Gene 1 duplex 1	Gene 1 duplex 3	Gene 5 duplex 1	Gene 13 duplex 1	Gene 7 duplex 2	Mock	Gene 15 duplex 2	Gene 9 duplex 3	Gene 17 duplex 3	Gene 11 duplex 3	Mock
B	Mock	Gene 2 duplex 1	Gene 2 duplex 3	Gene 6 duplex 1	Gene 14 duplex 1	Gene 8 duplex 2	Mock	Gene 16 duplex 2	Gene 10 duplex 3	Gene 18 duplex 3	Gene 12 duplex 4	Mock
C	Mock	Gene 3 duplex 1	Gene 3 duplex 3	Gene 7 duplex 1	Gene 15 duplex 1	Gene 9 duplex 2	Mock	Gene 17 duplex 2	Gene 11 duplex 3	Gene 5 duplex 4	Gene 13 duplex 4	Mock
D	si_CDC42	Gene 4 duplex 1	Gene 4 duplex 3	Gene 8 duplex 1	Gene 16 duplex 1	Gene 10 duplex 2	Mock	Gene 18 duplex 2	Gene 12 duplex 3	Gene 6 duplex 4	Gene 14 duplex 4	si_CDC42
E	si_CDH5	Gene 1 duplex 2	Gene 1 duplex 4	Gene 9 duplex 1	Gene 17 duplex 1	Gene 11 duplex 2	Mock	Gene 5 duplex 3	Gene 13 duplex 3	Gene 7 duplex 4	Gene 15 duplex 4	si_CDH5
F	Mock	Gene 2 duplex 2	Gene 2 duplex 4	Gene 10 duplex 1	Gene 18 duplex 1	Gene 12 duplex 2	Mock	Gene 6 duplex 3	Gene 14 duplex 3	Gene 8 duplex 4	Gene 16 duplex 4	Mock
G	Mock	Gene 3 duplex 2	Gene 3 duplex 4	Gene 11 duplex 1	Gene 5 duplex 2	Gene 13 duplex 2	Mock	Gene 7 duplex 3	Gene 15 duplex 3	Gene 9 duplex 4	Gene 17 duplex 4	Mock
H	Mock	Gene 4 duplex 2	Gene 4 duplex 4	Gene 12 duplex 1	Gene 6 duplex 2	Gene 14 duplex 2	Mock	Gene 8 duplex 3	Gene 16 duplex 3	Gene 10 duplex 4	Gene 18 duplex 4	Mock

Tertiary screen plate layout

	1	2	3	4	5	6	7	8	9	10	11	12
A	Mock	siRNA SMARTpools	siRNA SMARTpools	siRNA SMARTpools	siRNA SMARTpools	siRNA SMARTpools	siRNA SMARTpools	siRNA SMARTpools	siRNA SMARTpools	siRNA SMARTpools	siRNA SMARTpools	Mock
B	Mock	siRNA SMARTpools	siRNA SMARTpools	siRNA SMARTpools	siRNA SMARTpools	siRNA SMARTpools	siRNA SMARTpools	siRNA SMARTpools	siRNA SMARTpools	siRNA SMARTpools	siRNA SMARTpools	Mock
C	si_RHOA	siRNA SMARTpools	siRNA SMARTpools	siRNA SMARTpools	siRNA SMARTpools	siRNA SMARTpools	siRNA SMARTpools	siRNA SMARTpools	siRNA SMARTpools	siRNA SMARTpools	siRNA SMARTpools	si_RHOA
D	si_CDH5	siRNA SMARTpools	siRNA SMARTpools	siRNA SMARTpools	siRNA SMARTpools	siRNA SMARTpools	siRNA SMARTpools	siRNA SMARTpools	siRNA SMARTpools	siRNA SMARTpools	siRNA SMARTpools	si_CDH5
E	si_CDC42	siRNA SMARTpools	siRNA SMARTpools	siRNA SMARTpools	siRNA SMARTpools	siRNA SMARTpools	siRNA SMARTpools	siRNA SMARTpools	siRNA SMARTpools	siRNA SMARTpools	siRNA SMARTpools	si_CDC42
F	Non-targeting #2	siRNA SMARTpools	siRNA SMARTpools	siRNA SMARTpools	siRNA SMARTpools	siRNA SMARTpools	siRNA SMARTpools	siRNA SMARTpools	siRNA SMARTpools	siRNA SMARTpools	siRNA SMARTpools	Non-targeting #2
G	Mock	siRNA SMARTpools	siRNA SMARTpools	siRNA SMARTpools	siRNA SMARTpools	siRNA SMARTpools	siRNA SMARTpools	siRNA SMARTpools	siRNA SMARTpools	siRNA SMARTpools	siRNA SMARTpools	Mock
H	Mock	siRNA SMARTpools	siRNA SMARTpools	siRNA SMARTpools	siRNA SMARTpools	siRNA SMARTpools	siRNA SMARTpools	siRNA SMARTpools	siRNA SMARTpools	siRNA SMARTpools	siRNA SMARTpools	Mock

Supplementary Figure 1. Plate layout of control and library siRNAs in Primary, Secondary deconvolution and Tertiary siRNA screens. Note that in the Secondary deconvolution plate the four colours (pale blue - dark blue) indicate the four siRNA duplex per gene.

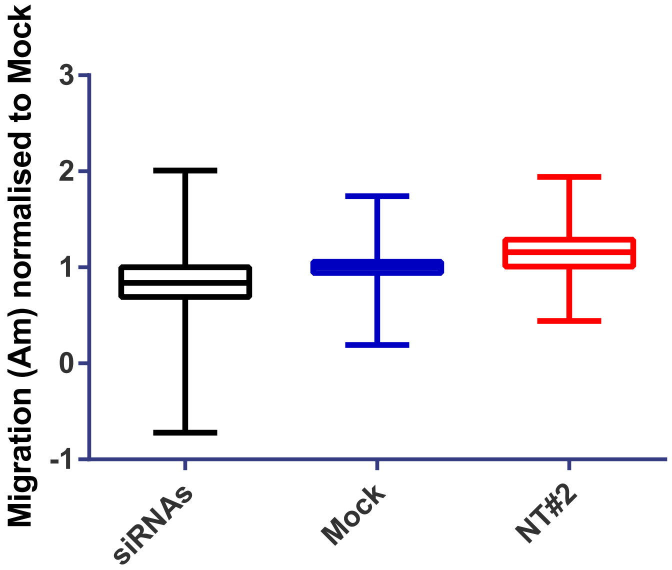

Supplementary Figure 2. Non-targeting siRNA #2 caused accelerated migration compared to Mock-transfected wells. The migration (Am) for each siRNA type is normalised to the mean of Mock-transfected wells. ‘siRNAs’ represents all siRNAs in the genome-wide siRNA library, excluding those that were scored as ‘Low Cell Count’ due to low viability. Boxplots show the median, upper and lower quartiles, and whiskers extending to minimum and maximum values.

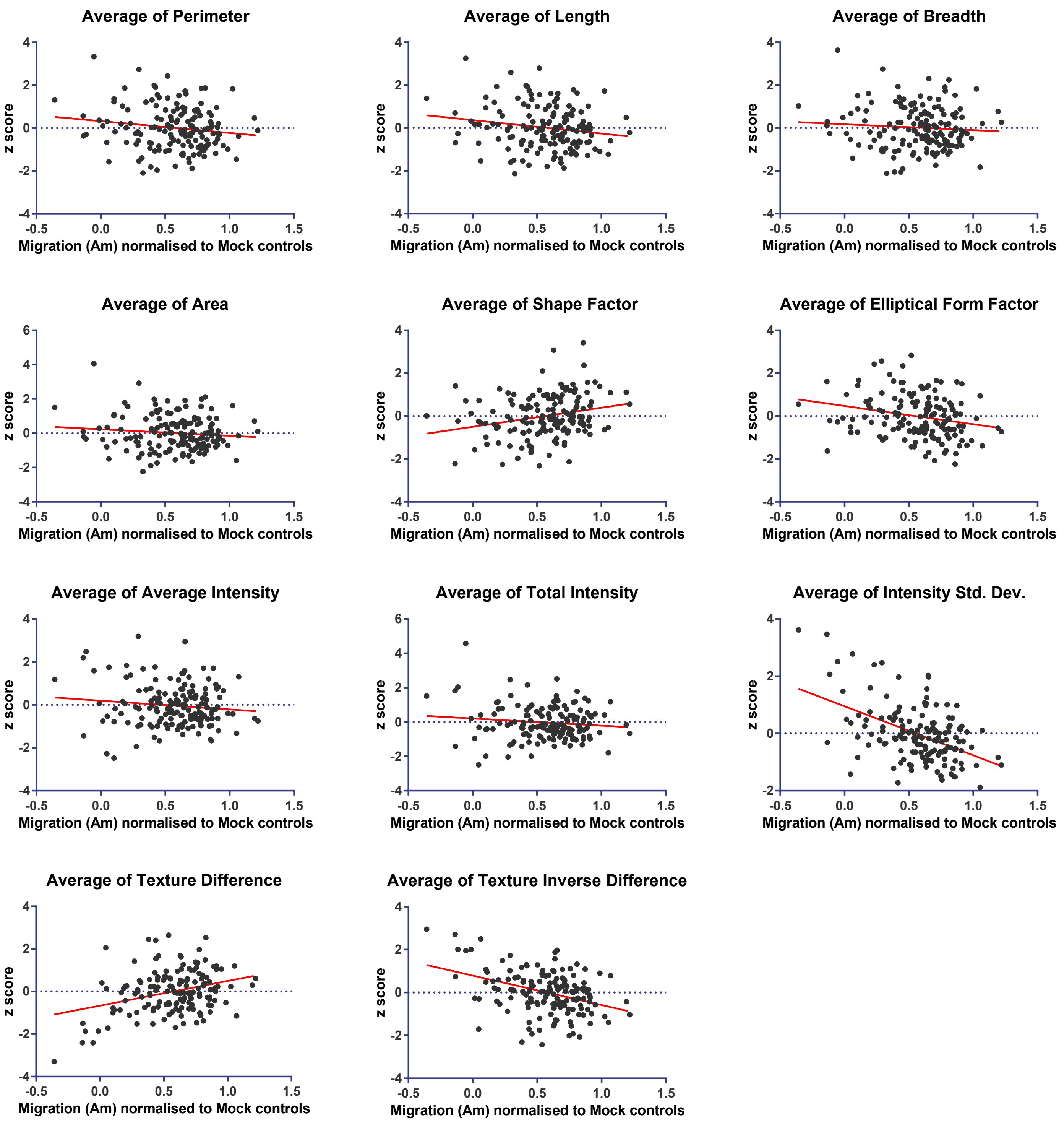

Supplementary Figure 3. Correlation between HDLEC normalised migration scores (Tertiary screen) and morphometric data from morphology analysis. The *y* axis on each graph represents the z-scored values for the indicated morphometric feature. Each point represents an siRNA-transfected population of cells. Red line indicates the linear regression fit line. Average Intensity, Total Intensity, Intensity Std. Dev., Texture Difference and Texture Inverse Difference refer to Phalloidin CF555 staining.

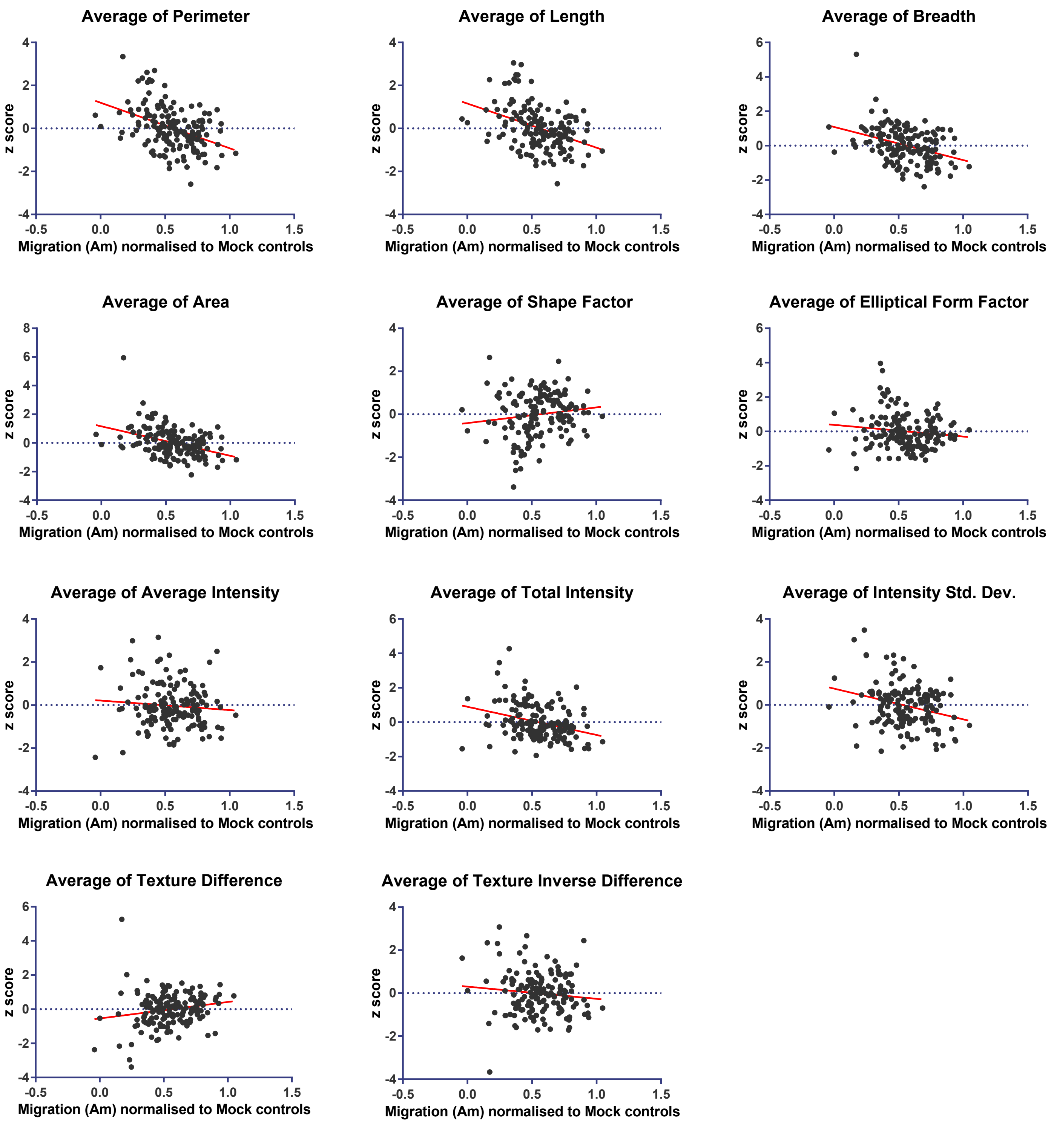

Supplementary Figure 4. Correlation between HMBEC normalised migration scores (Tertiary screen) and morphometric data from morphology analysis. The *y* axis on each graph represents the z-scored values for the indicated morphometric feature. Each point represents an siRNA-transfected population of cells. Red line indicates the linear regression fit line. Average Intensity, Total Intensity, Intensity Std. Dev., Texture Difference and Texture Inverse Difference refer to Phalloidin CF555 staining.
